# Comparative analysis of clinical, patient-centered, and COL1A1 gene expression outcomes following coronally advanced flap with xenogeneic collagen matrix versus connective tissue graft in maxillary gingival recession: A parallel-arm, single-blinded randomized controlled clinical trial

**DOI:** 10.1016/j.jobcr.2025.12.010

**Published:** 2025-12-17

**Authors:** Vazeeha Afrin Syed, Arvina Rajasekar

**Affiliations:** Department of Periodontology, Saveetha Dental College and Hospitals, Saveetha Institute of Medical and Technical Sciences (SIMATS), Saveetha University, Chennai, India

**Keywords:** Autogenous graft, Collagen matrix, Gingival recession, Recession, Regeneration

## Abstract

**Objectives:**

Coronally advanced flap (CAF) combined with connective tissue graft (CTG) is a gold standard for root coverage but is limited by donor site morbidity. The aim of this study was to compare the clinical, patient-centered, and molecular outcomes of CAF combined with xenogeneic collagen matrix (XCM) versus CTG in the treatment of maxillary gingival recession defects, with a focus on COL1A1 gene expression.

**Methods:**

This randomized controlled trial included 40 patients with Cairo RT1 gingival recession defects, allocated into two groups: CAF + XCM (test; n = 20) and CAF + CTG (control; n = 20). Clinical parameters including probing pocket depth (PPD), clinical attachment level (CAL), width of keratinized tissue (WKT), recession height (RH), recession width (RW), and mean root coverage (MRC) were evaluated at baseline and 6 months. Root sensitivity was assessed at baseline and 6 months, while postoperative pain was recorded at 24 h, 7 days, and 14 days. Surgical duration was measured, and COL1A1 gene expression in gingival crevicular fluid was quantified using real-time quantitative PCR.

**Results:**

Both groups showed significant clinical improvement and upregulation of COL1A1 expression at 6 months (p < 0.05). Intergroup differences in clinical and molecular outcomes were not statistically significant. However, the XCM group had significantly shorter surgical time and lower postoperative pain scores (p < 0.05), indicating improved patient comfort.

**Conclusions:**

XCM offers comparable clinical and molecular outcomes to CTG with the added benefits of reduced surgical time and morbidity, making it a viable, patient-friendly alternative in the management of gingival recession.

## 1 Introduction

Gingival recession is a prevalent periodontal condition characterized by the apical displacement of the gingival margin from the cemento-enamel junction (CEJ), exposing the root surface. Its etiology is multifactorial, involving a combination of periodontal diseases, anatomical vulnerabilities, and traumatic oral habits. This condition not only poses aesthetic concerns but also contributes to dentin hypersensitivity and an elevated risk of root surface caries.^1^ Conventional therapeutic interventions have predominantly revolved around autogenous tissue grafting techniques.^2^ However, the advent of regenerative medicine has propelled the exploration of novel biomaterials aimed at enhancing periodontal tissue restoration.^3^, ^4^, ^5^, ^6^, ^7^, ^8^, ^9^

Among the traditional approaches, connective tissue grafting (CTG) remains a cornerstone for the surgical correction of gingival recession.^10^ The connective tissue plays a critical role in maintaining the functional and structural integrity of the periodontium. It is composed of a cellular component embedded within an extracellular matrix, in which collagen serves as the principal structural protein. Various collagen types, including I, III, IV, V, and VI, are distributed throughout the periodontal apparatus, each contributing to distinct tissue properties. Type I collagen is the predominant form found in both alveolar bone and gingival connective tissue, reflecting its importance in tissue resilience and repair.^11^ The COL1A1 gene encodes the alpha-1 chain of type I collagen, and its expression is integral to the synthesis, organization, and maintenance of the extracellular matrix. Aberrations in COL1A1 gene expression, whether due to genetic mutations or pathological regulation, have been implicated in connective tissue dysfunction and alveolar bone resorption, ultimately affecting periodontal stability.^12^

While CTG is highly efficacious and facilitates keratinized tissue formation—owing to its capacity to biologically influence the overlying epithelium—it is not without limitations. Common drawbacks include donor site morbidity, restricted graft availability, the necessity for a secondary surgical site, and the risk of postoperative graft contraction.^13^ In light of these limitations, recent advances have focused on the development of biomaterials that mitigate the need for autogenous harvesting while preserving or enhancing therapeutic efficacy.^14^

Xenogeneic collagen matrices (XCM) have emerged as a promising biomaterial alternative in periodontal regenerative therapy. Derived from non-human sources such as bovine or porcine tissues, XCMs are engineered to function as scaffolds that support cellular attachment, proliferation, and differentiation, thereby promoting tissue regeneration. Their inherent biocompatibility and reduced immunogenicity—achieved through meticulous processing to eliminate antigenic components—make them suitable for clinical application.^15^ XCMs are now widely employed in root coverage surgeries, where they offer the dual benefit of biological integration and reduced patient morbidity.^16^

Among commercially available XCMs, Fibro-Gide® has gained considerable attention due to its favorable biological and mechanical properties. Composed primarily of porcine-derived collagen, Fibro-Gide® is specifically engineered to support fibroblast activity, which is crucial for the regeneration of connective tissues in the periodontal environment. When applied to recession defects, it serves as a scaffold that facilitates fibroblast migration and extracellular matrix formation, thereby supporting the regeneration of lost connective tissue. Its versatility in size and thickness allows for customization to a variety of defect morphologies, enhancing its clinical applicability. Additionally, Fibro-Gide® contributes to clot stabilization and offers protective coverage during healing, minimizing the risk of postoperative complications and enhancing treatment outcomes.^17^

This study aims to evaluate the clinical and patient-centered outcomes associated with the use of Fibro-Gide® in comparison to connective tissue grafting when combined with the coronally advanced flap (CAF) technique for the treatment of gingival recession in maxillary teeth. Furthermore, it investigates the underlying molecular mechanisms of periodontal regeneration by assessing the expression of the COL1A1 gene. Through this dual focus on clinical efficacy and molecular biology, the study seeks to enrich current understanding of regenerative strategies in periodontal therapy and to validate the therapeutic potential of XCM in soft tissue management.

## 2 Materials and methods

### 2.1 Study design

**Fig. 1:**
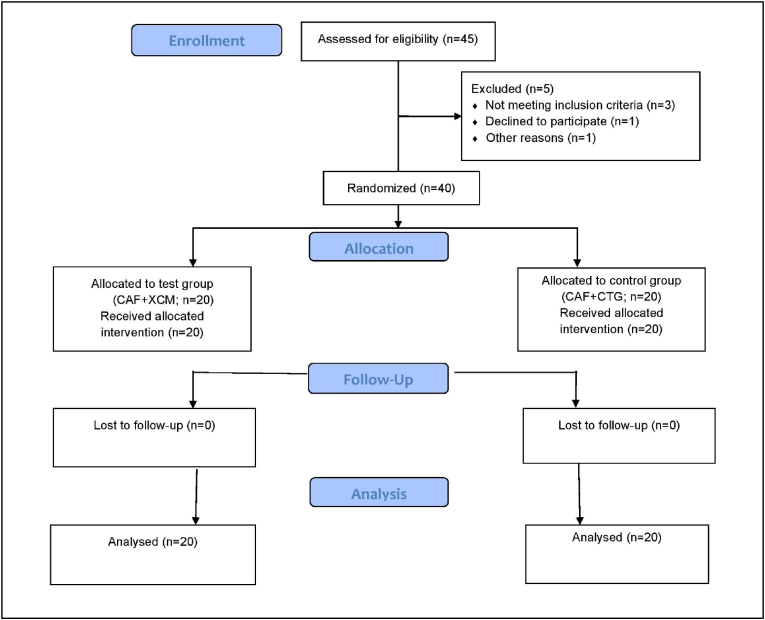
Consort flow chart.

This parallel-arm, single-blinded randomized controlled clinical trial was conducted from July 2024 to November 2024 at the Department of Periodontology, Saveetha University. Ethical clearance was obtained from the Institutional Human Ethical Committee (IHEC/SDC/PERIO-2201/24/025), and the trial was conducted in adherence to the ethical principles outlined in the 2013 revision of the Declaration of Helsinki. The study design (Fig. 1) followed CONSORT guidelines (http://www.consort-statement.org/) and was registered in the Clinical Trials Registry of India (CTRI/2024/06/068420). Written informed consent was obtained from all participants before enrollment. A total of 40 individuals reporting to the outpatient clinic of the Periodontics Department, Saveetha Dental College and Hospitals, Chennai, were recruited after providing informed consent and meeting the eligibility criteria.

#### 2.1.1 Study population

Patients seeking treatment for gingival recession were screened and enrolled based on the following criteria:

Inclusion Criteria:

1. Adults aged between 25 and 50 years.
2. Subjects with a plaque index (PI)^18^ of 0.1–0.9 and gingival index (GI)^18^ of 0.1–1.
3. Recession type RT1 (Cairo classification), with intact interproximal attachment and CEJ detectable from both proximal surfaces.
4. Presence of gingival recession in at least two adjacent maxillary non-molar teeth.
5. Systemically healthy individuals.
6. Teeth with probing depths ≤3 mm and no signs of periodontitis.

Exclusion Criteria:

1. Presence of systemic diseases.
2. Patients diagnosed with periodontitis.
3. Chronic medication usage.
4. Pregnant or lactating women.
5. Known allergy or hypersensitivity to xenogeneic collagen matrices.
6. Tobacco users or smokers.
7. Prior mucogingival or periodontal surgery at the treatment site.
8. Presence of carious or non-carious cervical lesions (NCCLs) at recession sites. NCCLs were evaluated clinically using visual inspection and tactile exploration with a periodontal probe (UNC-15, Hu-Friedy®, USA) under adequate illumination. Lesions presenting loss of cervical tooth structure with definable saucer-shaped or wedge-shaped morphology due to abrasion, erosion, or abfraction were identified as NCCLs and excluded.

### 2.2 Sample size calculation

Sample size was calculated using G∗Power software (Version 3.1.9.4) with 80 % power and a 95 % confidence interval, based on recession height values reported in previous literature.^19^ A total of 40 patients were randomized equally into two groups: Test group (CAF + XCM, n = 20) and Control group (CAF + CTG, n = 20).

### 2.3 Randomization, allocation concealment, and blinding

Randomization was performed using a simple computerized method (RandomAlloc.exe). Allocation was concealed using sealed, opaque, coded envelopes, which were opened only after flap elevation during surgery. The outcome assessor (AR) remained blinded to the group allocation throughout the study.

### 2.4 Intervention

All patients underwent a thorough periodontal evaluation by a single calibrated examiner (AR). Oral hygiene instructions emphasizing non-traumatic brushing techniques were provided, and professional prophylaxis (scaling and polishing) was performed preoperatively.

**Fig. 2:**
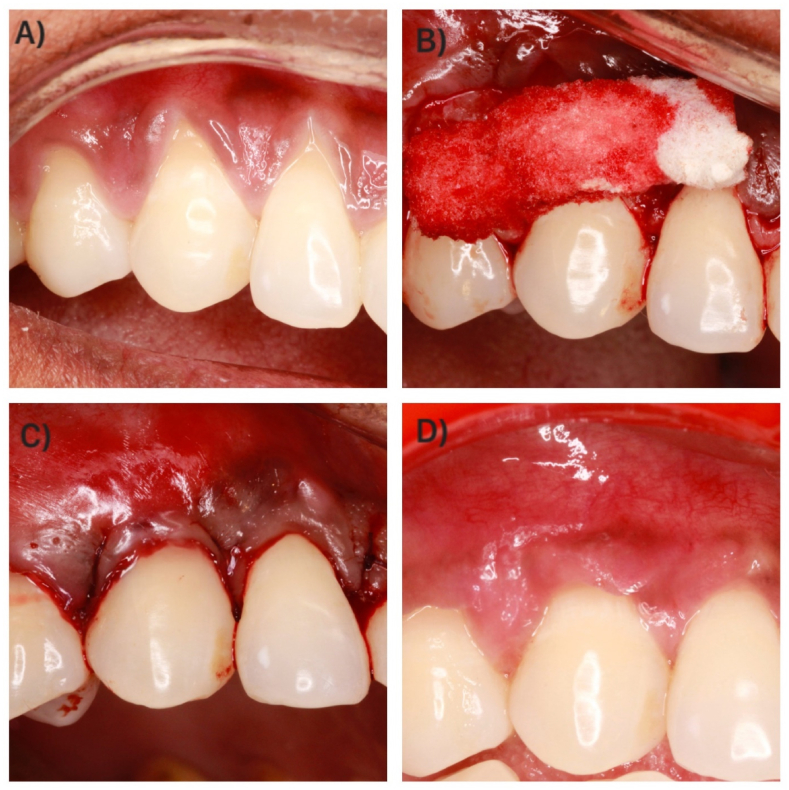
Surgical procedure in test group. A - Clinical presentation at baseline. B - Fibro-Gide® placement. C - Flap sutured with interrupted sling suture. D - Clinical presentation at 6 months follow-up.

**Fig. 3:**
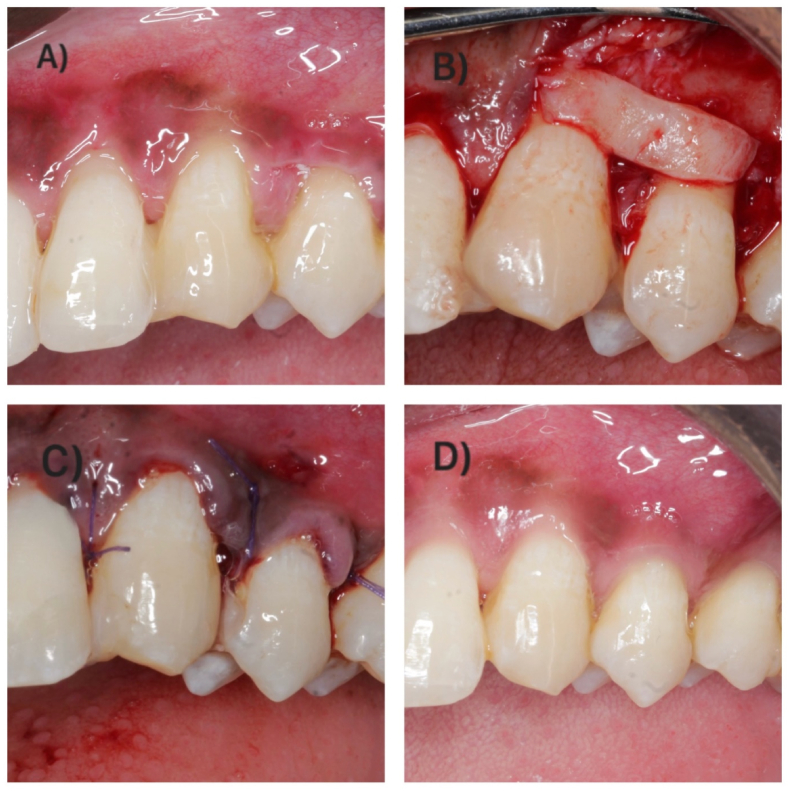
Surgical procedure in control group. A - Clinical presentation at baseline. B - CTG placement. C - Flap sutured with interrupted sling suture. D - Clinical presentation at 6 months follow-up.

Surgical procedures were carried out by a single clinician (VAS). A split-full-split thickness flap was reflected without vertical releasing incisions, as per the technique described by Zucchelli and De Sanctis.^20^ Following flap elevation, root surfaces were debrided using Gracey curettes (Hu-Friedy®, Chicago, USA). Treatment allocation was then revealed. In the test group (Fig. 2), XCM (Fibro-Gide®, Geistlich, USA) was placed over the recession defect per the manufacturer's protocol. In the control group (Fig. 3), CTG was harvested from the palate following Harris's technique.^21^ Both the CTG and XCM were stabilized over the interdental papillae using simple interrupted sutures with resorbable material (Vicryl 5-0, Ethicon Inc., NJ, USA). Subsequently, the flap was coronally advanced and secured 1–2 mm above the CEJ with interrupted sling sutures using resorbable material (Vicryl 5-0, Ethicon Inc., NJ, USA). All patients received analgesics (Zerodol-P) postoperatively and were prescribed chlorhexidine mouthwash (0.12 %) twice daily for 14 days. Brushing was avoided in the surgical area for two weeks, after which ultra-soft brushes were introduced. Patients were reassessed at 24 h, 7 days, and 14 days postoperatively.

### 2.5 Outcome measures

Clinical Parameters:

1. Probing Pocket Depth (PPD)
2. Clinical Attachment Level (CAL)
3. Width of Keratinized Tissue (WKT)
4. Recession Height (RH)
5. Recession Width (RW)
6. Mean Root Coverage (MRC)

Patient-Centered Outcomes:

1. Pain assessment
2. Root sensitivity

Additional Parameters:

1. Surgical duration (in minutes)
2. Expression level of the COL1A1 gene

### 2.6 Examiner calibration

All clinical measurements were recorded by a single calibrated examiner (AR). To assess intra-rater reliability, 10 gingival recession sites were evaluated at two separate time points, spaced 2 h apart. The intra-class correlation coefficient (ICC) was found to be 0.89 for RH (95 % CI: 0.84–0.94) and 0.79 for RW (95 % CI: 0.68–0.86), indicating a high level of measurement consistency.

### 2.7 Periodontal measurements

A custom acrylic stent was fabricated using stone casts of maxillary arches. A standardized groove on the mid-buccal surface allowed reproducible placement of the periodontal probe (UNC-15, Hu-Friedy®, USA). Baseline and 6-month post-surgical measurements included:

• PPD: Distance from gingival margin to base of the sulcus
• CAL: Distance from CEJ to base of sulcus
• WKT: Distance from gingival margin to mucogingival junction
• RH: Distance from CEJ to gingival margin
• RW: Horizontal span of the recession at CEJ level
• MRC (%): [(RH at baseline - RH at 6 months)/RH at baseline] × 100
• PI^18^
• GI^18^

### 2.8 Surgical time

Measured from the initial incision to the final suture placement, in minutes.

### 2.9 Patient-reported outcomes

Pain and root sensitivity were assessed using a Visual Analog Scale (VAS) ranging from 0 (no pain/sensitivity) to 10 (severe pain/sensitivity). Root sensitivity was measured using a controlled air-blast at baseline and 6 months post-surgery. Pain was recorded at 24 h, 7 days, and 14 days postoperatively.

### 2.10 Sample collection and gene expression analysis of COL1A1

Gingival crevicular fluid (GCF) was collected from surgical sites at baseline and 6 months using calibrated microcapillary pipettes after isolation with sterile cotton rolls. Samples were stored in RNase-free tubes at −80 °C until RNA extraction using RNAiso Plus reagent (Takara Bio Inc., Japan). RNA quantity and purity were assessed with a Synergy H1 Microplate Reader (BioTek, USA), accepting samples with an A260/A280 ratio of 1.8–2.0. RNA integrity was verified via agarose gel electrophoresis. cDNA was synthesized from 1 μg RNA using the PrimeScript™ RT reagent kit (Takara Bio Inc., USA), which included a genomic DNA removal step. Real-time quantitative PCR (RT-qPCR) was performed using the CFX Opus 96 System (Bio-Rad, USA) and Taq Universal SYBR Green Supermix (Bio-Rad, USA). Primer sequences were:

• COL1A1:
  ○ Forward: 5′-GTSCTAAAGGTGCCAATGGT-3′
  ○ Reverse: 5′-ACCAGGTTCACCGCTGTTAC-3′
• GAPDH (Reference gene):
  ○ Forward: 5′-AACAGCGACACCCACTCCTC-3′
  ○ Reverse: 5′-CATACCAGGAAATGAGCTTGACAA-3′

The thermal profile included denaturation at 95 °C for 3 min, followed by 30 cycles of 95 °C for 10 s and 60 °C for 10 s. A melt curve from 65 °C to 95 °C confirmed amplification specificity. Relative gene expression was quantified using the 2^−ΔΔCt^ method.

### 2.11 Statistical analysis

Data analysis was conducted using SPSS software (Version 23.0; IBM Corp., Armonk, NY, USA). Normality was assessed using the Shapiro-Wilk test. Quantitative data were presented as mean ± standard deviation. Age differences were evaluated using the independent *t*-test, while gender and site-wise distributions were compared using the chi-square test. Normally distributed parameters (PPD, CAL, RH, RW, KTW, PI, GI, and COL1A1) were analyzed using paired t-tests for intra-group comparisons and independent t-tests for inter-group comparisons. Root sensitivity scores, being non-parametric, were assessed using the Wilcoxon signed-rank test (intra-group) and the Mann–Whitney *U* test (inter-group). Postoperative pain was evaluated at 24 h, 7 days, and 14 days using the Friedman test for intra-group comparisons and the Mann–Whitney *U* test for inter-group analysis at each time point. Surgical time and MRC were also compared using independent t-tests. A p-value <0.05 was considered statistically significant.

## 3 Results

The demographic characteristics of participants in both the CAF + XCM and CAF + CTG groups revealed no significant differences. The mean age was 39.14 ± 6.54 years in the CAF + XCM group and 38.32 ± 7.35 years in the CAF + CTG group (p = 0.711), indicating comparability in age distribution. The gender ratio was similar across groups (Male/Female: 11/9 in XCM, 10/10 in CTG; p = 1.01). Site-wise distribution of treated teeth in the maxilla was also statistically similar between the groups (p = 0.99), ensuring baseline homogeneity and eliminating demographic confounders (Table 1).

**Table 1:** Demographic characteristics of participants in the CAF + XCM and CAF + CTG groups.

Parameters		CAF + XCM	CAF + CTG	p value
Age		39.14 ± 6.54	38.32 ± 7.35	0.711
Gender	Male/Female	11/9	10/10	1.01
Site (Maxilla)	Central Incisor	3	3	0.99
	Lateral Incisor	9	9	
	Canine	12	12	
	1st Premolar	12	12	
	2nd Premolar	17	18	
	Total	53	54	

Table 2 shows no statistically significant intergroup differences at baseline and 6 months between CAF + XCM and CAF + CTG groups across all parameters. At baseline, values for PPD (*p* = 0.754), CAL (*p* = 0.460), RH (*p* = 0.757), RW (*p* = 0.846), WKT (*p* = 0.770), PI (*p* = 0.630), GI (*p* = 0.849), COL1A1 expression (*p* = 0.875), and sensitivity scores (*p* = 0.190) were comparable. At 6 months, clinical improvements were observed in both groups, yet differences remained non-significant for PPD (*p* = 0.321), CAL (*p* = 0.268), RH (*p* = 0.105), RW (*p* = 0.517), WKT (*p* = 0.804), MRC (*p* = 0.706), PI (*p* = 0.747), GI (*p* = 0.472), COL1A1 expression (*p* = 0.820), and sensitivity (*p* = 0.114), indicating both interventions yielded comparable clinical and molecular outcomes.

**Table 2:** Inter-group comparison at baseline and 6-month follow-up between CAF + XCM and CAF + CTG cohorts.

Parameters	Baseline		p value	6 months		p value
	CAF + XCM	CAF + CTG		CAF + XCM	CAF + CTG	
PPD	1.29 ± 0.29	1.32 ± 0.31	0.754^a^	2.54 ± 0.11	2.58 ± 0.14	0.321^a^
CAL	4.21 ± 0.44	4.32 ± 0.49	0.460^a^	2.81 ± 0.08	2.83 ± 0.09	0.268^a^
WKT	2.41 ± 0.21	2.39 ± 0.22	0.770^a^	3.73 ± 0.49	3.69 ± 0.52	0.804^a^
RH	3.11 ± 0.42	3.15 ± 0.39	0.757^a^	0.46 ± 0.09	0.51 ± 0.10	0.105^a^
RW	4.27 ± 0.87	4.32 ± 0.74	0.846^a^	1.06 ± 0.15	1.09 ± 0.14	0.517^a^
MRC	–	–	–	85.21 ± 11.08	83.81 ± 12.19	0.706^a^
PI	0.45 ± 0.12	0.43 ± 0.14	0.630^a^	0.105 ± 0.03	0.102 ± 0.03	0.747^a^
GI	0.56 ± 0.17	0.55 ± 0.16	0.849^a^	0.17 ± 0.04	0.16 ± 0.04	0.472^a^
Relative COL1A1 mRNA expression level	4.35 ± 0.48	4.38 ± 0.52	0.875^a^	3.08 ± 0.49	3.12 ± 0.45	0.820^a^
Sensitivity	5.04 ± 1.06	5.08 ± 1.1	0.190^b^	1.09 ± 0.12	1.11 ± 0.08	0.114^b^

a Independent *t*-test.

b Mann Whitney *U* test.

Intragroup comparisons (Table 3) demonstrated statistically significant improvements from baseline to 6 months in both groups (p < 0.05 for all parameters). Furthermore, a significant decline in ΔCt values for COL1A1 was observed within each group—CAF + XCM (p = 0.002) and CAF + CTG (p = 0.001)—implying an upregulation of gene expression post-treatment. These results affirm the regenerative potential of both interventions at clinical and molecular levels, supporting their use in managing gingival recession defects.

**Table 3:** Intra-group changes from baseline to 6 months post-treatment in both CAF + XCM and CAF + CTG groups.

Parameters	CAF + XCM		p value	CAF + CTG		p value
	Baseline	6 months		Baseline	6 months	
PPD	1.29 ± 0.29	2.54 ± 0.11	0.000^a^∗	1.32 ± 0.31	2.58 ± 0.14	0.000^a^∗
CAL	4.21 ± 0.44	2.81 ± 0.08	0.000^a^∗	4.32 ± 0.49	2.83 ± 0.09	0.000^a^∗
WKT	2.41 ± 0.21	3.73 ± 0.49	0.000^a^∗	2.39 ± 0.22	3.69 ± 0.52	0.000^a^∗
RH	3.11 ± 0.42	0.46 ± 0.09	0.000^a^∗	3.15 ± 0.39	0.51 ± 0.10	0.000^a^∗
RW	4.27 ± 0.87	1.06 ± 0.15	0.000^a^∗	4.32 ± 0.74	1.09 ± 0.14	0.000^a^∗
PI	0.45 ± 0.12	0.105 ± 0.03	0.000^a^∗	0.43 ± 0.14	0.102 ± 0.03	0.000^a^∗
GI	0.56 ± 0.17	0.17 ± 0.04	0.000^a^∗	0.55 ± 0.16	0.16 ± 0.04	0.000^a^∗
Relative COL1A1 mRNA expression level	4.35 ± 0.48	3.08 ± 0.49	0.002^a^∗	4.38 ± 0.52	3.12 ± 0.45	0.001^a^∗
Sensitivity	5.04 ± 1.06	1.09 ± 0.12	0.000^b^∗	5.08 ± 1.1	1.11 ± 0.08	0.000^b^∗

∗ – Statistically significant (p < 0.05).

a Paired *t*-test.

b Wilcoxon signed rank test.

The mean surgical time was significantly shorter in the CAF + XCM group (36.23 ± 9.21 min) compared to the CAF + CTG group (52.13 ± 12.16 min), with a highly significant p-value (p = 0.000). This finding suggests that XCM offers a time-efficient alternative to autogenous graft harvesting, potentially reducing surgical morbidity and chair time (Table 4).

**Table 4:** Comparative analysis of surgical time required for procedures involving CAF + XCM and CAF + CTG.

Parameters	CAF + XCM	CAF + CTG	p value
Surgical time (mins)	36.23 ± 9.21	52.13 ± 12.16	0.000^a^∗

∗ – Statistically significant (p < 0.05).

a Independent *t*-test.

Postoperative pain assessment revealed significantly lower pain scores in the CAF + XCM group at all measured time points—24 h (2.45 ± 0.67 vs. 6.12 ± 1.13), 7 days (1.23 ± 0.36 vs. 3.82 ± 0.23), and 14 days (0.45 ± 0.01 vs. 1.22 ± 0.14)—compared to the CAF + CTG group (all p = 0.000). This trend underscores the improved patient comfort and reduced morbidity associated with the XCM procedure over the conventional CTG technique (Table 5).

**Table 5:** Evaluation of postoperative pain at 24 h, 7 days, and 14 days between CAF + XCM and CAF + CTG groups.

Groups	24 h	7 days	14 days	p value
CAF + XCM	2.45 ± 0.67	1.23 ± 0.36	0.45 ± 0.01	0.000^a^∗
CAF + CTG	6.12 ± 1.13	3.82 ± 0.23	1.22 ± 0.14	0.000^a^∗
p value	0.000^b^∗	0.000^b^∗	0.000^b^∗	

∗ – Statistically significant (p < 0.05).

a Friedman test.

b Mann Whitney *U* test.

## 4 Discussion

The treatment of gingival recession remains a significant challenge in periodontal surgery, with various approaches evaluated for their clinical efficacy. Among these, CAF combined with CTG has been considered the gold standard. However, CTG carries disadvantages such as donor site morbidity and increased surgical time. To mitigate these issues, the use of XCM has emerged as a promising alternative for periodontal tissue regeneration. In this study, we aimed to compare the clinical, patient-centered outcomes and COL1A1 gene expression of XCM versus CTG in the treatment of gingival recession, with the goal of providing further insights into their relative efficacy and safety.

The results of our study showed that both XCM and CTG led to significant improvements in clinical parameters such as PPD, CAL, WKT, RH, RW, MRC, and root sensitivity. However, XCM was associated with significantly reduced postoperative pain till two weeks compared to CTG. Additionally, the use of XCM resulted in shorter surgical time, which could be beneficial for patient comfort and surgical efficiency. These findings are in line with the study by Gürlek et al.,^22^ who compared the clinical efficacy of XCM and CTG in combination with a modified coronally advanced flap (M-CAF) for treating multiple gingival recessions. The study showed no significant differences in RH, RW, or WKT at 18 months, confirming that XCM can serve as an alternative to CTG without compromising clinical outcomes. Similarly, Meza-Mauricio et al.^23^ demonstrated that both CAF + CTG and CAF + XCM resulted in comparable root coverage and gingival thickness, with the CAF + XCM group offering reduced postoperative pain and shorter recovery times. These studies underscore the potential of XCM as a suitable alternative to CTG, especially in cases where reduced morbidity and faster healing are desired.

Jepsen et al.^24^ further reinforced these findings, showing that adding XCM to CAF resulted in enhanced gingival thickness and WKT compared to CAF alone. However, no significant difference in root coverage was observed between the two treatment modalities. This is consistent with our study, where both XCM and CTG produced similar improvements in clinical outcomes, but the XCM group exhibited a reduction in patient morbidity. Our study also corroborates the findings by Tonetti MS et al.,^25^ who reported a 36-month follow-up of a trial comparing the adjunct of XCM or CTG with CAF for the coverage of multiple adjacent recessions. The study found that while CTG had a slightly superior root coverage (2.0 ± 1.0 mm) compared to XCM (1.5 ± 1.5 mm), no significant differences were observed between the groups, indicating that XCM may offer comparable outcomes to CTG in terms of gingival margin position. In addition, the study by Hamid AM et al.^26^ compared the clinical benefits and effectiveness of XCM to CTG for the treatment of gingival recession. Both treatment groups showed significant improvements in clinical parameters such as attached gingiva width, probing depth, clinical attachment loss, and gingival recession. While there were no significant differences between the two groups, patient satisfaction was higher in XCM group, suggesting that XCM offers advantages in terms of patient comfort. These findings further highlight the potential of XCM as an efficient and less invasive alternative to CTG, with improved patient satisfaction.

Long-term studies have provided additional insights into the durability of treatment outcomes with collagen matrices and CTG. Milinkovic I et al.^27^ assessed the long-term clinical outcomes of XCM and CTG in the management of multiple adjacent gingival recessions using a split-mouth design. The study demonstrated that both XCM and CTG resulted in stable clinical outcomes over a five-year period, with no significant differences in MRC, WKT, or CAL. Similarly, a study by Molnar B et al.^28^ conducted a nine-year follow-up on the long-term outcomes following treatment of multiple adjacent gingival recessions using modified coronally advanced tunnel (MCAT) in conjunction with either XCM or CTG. Although there was some relapse in root coverage, especially in the mandibular regions, the outcomes were more stable in the maxillary regions. The study concluded that both XCM and CTG, when used in combination with MCAT, are effective in the long term, though the maxillary areas showed more stable outcomes. Moreover, Skurska A et al.^29^ compared the long-term results of MCAT combined with XCM or CTG in the treatment of Cairo recession Type 1 in mandibular single-rooted teeth. The study showed that the CTG-treated sites had significantly better outcomes in terms of MRC and other clinical parameters compared to the XCM-treated sites. However, the five-year follow-up data demonstrated stable results for both treatments, suggesting that both XCM and CTG provide stable long-term clinical results. Also, a recent meta-analysis compared the effectiveness of collagen matrix and subepithelial connective tissue in root coverage procedures. Results from nine eligible studies indicated that subepithelial connective tissue achieved significantly better outcomes in mean root coverage and keratinized tissue width. However, collagen matrix combined with a coronally advanced flap showed comparable potential for achieving complete root coverage.^30^

From a molecular perspective, recent evidence suggests that XCM promotes fibroblast migration and enhances collagen deposition—both of which are vital for effective tissue regeneration and successful root coverage.^12^ Collagen, being a natural component of the human body, supports periodontal tissue regeneration. COL1A1, a gene encoding type I collagen, plays a pivotal role in maintaining tissue integrity and facilitating wound healing. Several studies have highlighted the importance of COL1A1 in both healthy periodontal tissues and in the pathogenesis of periodontal disease. Alterations in COL1A1 expression have been shown to affect tissue architecture and contribute to periodontal disease progression.^31^^,^^32^ For instance, Gajendrareddy et al.^33^ investigated how oxygen exposure impacts collagen expression in ligature-induced periodontitis and observed significant alterations in COL1A1 expression, reflecting changes in tissue architecture in response to inflammation. These findings underscore the gene's importance in periodontal health and disease dynamics. In the present study, both CTG and XCM resulted in upregulation of COL1A1 expression during the healing phase, suggesting that both treatment modalities facilitate a regenerative microenvironment conducive to collagen synthesis. Cheng and Shen^34^ also identified COL1A1 as a key gene implicated in the pathophysiology of periodontitis, highlighting its critical role in maintaining periodontal tissue homeostasis. Therapeutic interventions that stimulate COL1A1 expression may thus contribute to improved tissue remodeling and healing outcomes.

The present study observed a notable upregulation of COL1A1 gene expression at six months postoperatively in both the CTG and XCM groups. This molecular response underscores the integral role of type I collagen in periodontal wound healing and matrix stabilization. Both treatment modalities were shown to induce collagen synthesis and deposition, essential for the structural maturation and integration of grafted tissues. The observed upregulation of COL1A1 further indicates the regenerative potential of XCM and CTG through similar molecular pathways, reinforcing their clinical efficacy in promoting soft tissue healing.

In conclusion, the present study provides valuable insights into the ongoing discussion regarding the use of XCM as an alternative to CTG in the treatment of gingival recession. Both treatment modalities led to significant improvements in clinical parameters, with comparable outcomes in root coverage and soft tissue regeneration. However, XCM demonstrated additional advantages such as reduced postoperative discomfort and shorter surgical duration, making it a favorable option for patients seeking minimally invasive treatments. Therefore, the use of XCM in combination with the CAF technique presents a promising alternative to CTG, particularly in cases where patient comfort, reduced morbidity, and expedited healing are of concern. Future studies involving larger sample sizes and extended follow-up periods are essential to validate these findings and to further explore the long-term benefits of collagen matrices in periodontal regeneration. Additionally, future investigations should include protein-level validation—such as immunohistochemistry or ELISA for type I collagen—to confirm the molecular findings and elucidate the sustained regenerative impact of these therapeutic modalities.

## 5 Conclusion

Both XCM and CTG, when combined with the CAF technique, demonstrated significant clinical improvements and enhanced COL1A1 gene expression at 6 months postoperatively. While no statistically significant differences were observed between the groups in clinical or molecular outcomes, the study's sample size may limit the ability to detect smaller, clinically relevant differences. The XCM group showed significantly reduced surgical time and postoperative pain, indicating a more patient-friendly and time-efficient alternative to CTG. Therefore, XCM represents a promising substitute for CTG in treating maxillary gingival recession, offering favorable clinical outcomes with reduced morbidity, though larger studies are needed to confirm its comparative regenerative potential.

## CRediT authorship contribution statement

**Vazeeha Afrin Syed:** Writing - Original draft, Software, Methodology, Formal analysis, Data curation, Conceptualization, Visualization, Writing - review & editing. **Arvina Rajasekar:** Visualization, Writing - Original draft, Writing - review & editing, Validation, Supervision.

## Ethical clearance

The study protocol approval was obtained from the Ethical Board of Saveetha Dental College and Hospitals (IHEC/SDC/PERIO-2201/24/025). Additionally, ethical approval was obtained from the Clinical Trials Registry of India (CTRI/2024/06/068420).

## Patient's or Guardian's consent

Written informed consent was provided by all participants.

## Source of funding

This research did not receive any specific grant from funding agencies in the public, commercial or not for profit sectors.

## Declaration of competing interest

The authors declare no conflict of interest.
